# Altered adrenal and gonadal steroids biosynthesis in patients with burn injury

**DOI:** 10.1016/j.clinms.2016.10.002

**Published:** 2016-10-26

**Authors:** Maria Bergquist, Fredrik Huss, Filip Fredén, Göran Hedenstierna, Johanna Hästbacka, Alan L. Rockwood, Mark M. Kushnir, Jonas Bergquist

**Affiliations:** a Department of Medical Sciences, The Hedenstierna Laboratory, Uppsala University, Uppsala, Sweden; b Department of Surgical Sciences, Plastic Surgery, Uppsala University, Uppsala, Sweden; c Department of Surgical Sciences, Anaesthesiology and Intensive Care, Uppsala University, Uppsala, Sweden; d Uppsala Burn Center, Uppsala University Hospital, Uppsala, Sweden; e Intensive Care Medicine Department of Perioperative, Intensive Care and Pain Medicine, University of Helsinki and Helsinki University Hospital, Finland; f ARUP Institute for Clinical & Experimental Pathology, 500 Chipeta Way, Salt Lake City, UT 84108-1221, USA; g Department of Pathology, University of Utah School of Medicine, Salt Lake City, USA; h Department of Chemistry – BMC, Analytical Chemistry, Uppsala University, P.O. Box 599, SE-751 24 Uppsala, Sweden

**Keywords:** Burn, Trauma, Gonadal steroids, Sex steroids, Estrogen, Testosterone, DHEA, Androstenedione, LC-MS/MS

## Abstract

**Introduction:**

Burn injury inevitably leads to changes in the endogenous production of cytokines, as well as adrenal and gonadal steroids. Previous studies have reported gender-related differences in outcome following burn injury, which suggests that gonadal steroids may play a role. The aim of this study was to assess alterations in concentration of endogenous steroids in patients with burn injury.

**Methods:**

For this single-center, prospective descriptive study, high-sensitivity liquid chromatography tandem mass spectrometry (LC-MS/MS)-based steroid quantification was used to determine longitudinal profiles of the concentrations of endogenous steroids in plasma from sixteen adult male patients with burn injury (14.5–72% of total body surface area). Steroids were extracted from plasma samples and analyzed using multiple reaction monitoring acquisition, with electrospray ionization on a triple quadruple mass spectrometer. Total protein concentration was measured in the samples using spectrophotometry.

**Results:**

Steroid and total protein concentration distributions were compared to reference intervals characteristic of healthy adult men. Concentrations of the following steroids in plasma of burn injured patients were found to correlate positively to the area of the burn injury: cortisol (*r* = 0.84), corticosterone (*r* = 0.73), 11-deoxycortisol (*r* = 0.72), androstenedione (*r* = 0.72), 17OH-progesterone (*r* = 0.68), 17OH-pregnenolone (*r* = 0.64) and pregnenolone (*r* = 0.77). Concentrations of testosterone decreased during the acute phase and were up to ten-times lower than reference values for healthy adult men, while concentrations of estrone were elevated. By day 21 after injury, testosterone concentrations were increased in younger, but not older, patients. The highest concentrations of estrone were observed on day 3 after the injury and then declined by day 21 to concentrations comparable to those observed on the day of the injury.

**Conclusion:**

Burn injury alters endogenous steroid biosynthesis, with decreased testosterone concentrations and elevated estrone concentrations, during the first 21 days after the injury. Concentrations of glucocorticoids, progestagens and androgen precursors correlated positively with the area of burn injury. The finding of increased estrone following burn injury needs to be confirmed in a larger hypothesis-driven study.

## 1 Introduction

Severe burn injury is associated with high mortality and multiple organ failure [1]. The stress factors after burn injury are many and continuous. Large open wounds, dressing changes, mechanical ventilation, surgery and infection may all cause severe inflammation. In addition, a hypermetabolic state may follow with increased metabolic rate, peripheral insulin resistance, extensive protein wasting, lean body mass loss, and bone and muscle catabolism, often complicated with functional and structural alterations of essential organs [2], [3], [4]. The altered homeostasis after burn injury inevitably leads to changes in the circulating levels of cytokines, glucagon, catecholamines, and adrenal and gonadal steroids [5], [6]. Marked perturbations in HPA axis and elevated cortisol concentrations have also been observed in major burn injury [7], [8], [9], [10], [11]. Moreover, gender-specific differences in mortality following burn injury have been previously reported [12], [13]. Among burn trauma patients, premenopausal women have a survival advantage, as compared to men and postmenopausal women [14], [15]. In addition, gender and age differences have been documented in concentrations of circulating cytokine and multiple organ dysfunction syndrome (MODS), following polytrauma [16]. Physiologic concentrations of estrogens under normal conditions were suggested to be immune-stimulatory, while testosterone is thought to suppress immunity [17], [18]. Hypotestosteronemia, defined as total serum testosterone < 2.5 ng/mL [19], was commonly observed in critically ill men [20], [21], [22].

Previous studies have demonstrated differences in concentrations of gonadal steroids in burn patients, but have focused only on estradiol and testosterone, and have not examined the upstream intermediates and precursors of the steroid biosynthesis pathway, (e.g., estrone, androstenedione, DHEA, pregnenolone, 17OH-pregnenolone and 17OH-progesterone). Commercially available immunoassays were shown to be unreliable for quantification of testosterone when endogenous concentrations are expected to be low [23].

This study is a single-center, prospective descriptive study of male adult patients with burn injury with the aim of assessing changes in the biosynthesis of adrenal and gonadal steroids during the acute (0–3 days) and the sub-acute (7–21 days) phases following burn injury. To our knowledge, this is the first report to assess concentration variability for a panel of endogenous steroids in patients with burn injury, using high sensitivity LC-MS/MS methods.

## 2 Materials and methods

### 2.1 Patients

The study was approved by the Ethical Committee for Human Research in Uppsala, Sweden

(Dnr 2011/484). Patients were recruited between March 2012 and March 2013 from a larger study cohort in the Burn Center (BC) of Uppsala University Hospital, Sweden. Informed consent was obtained via next of kin and directly from survivors as soon as possible. Adult patients (>18 years) were eligible regardless of burn injury type. Exclusion criteria were malignancy, immune deficiency (HIV, cytostatic drugs, corticosteroids, tetracyclines or certain bisphosphonates), known or suspected blood-transmitted infections and participation in another clinical study within the last 4 weeks. Patients were partitioned into two subgroups based on burn size: severely injured (>20% burned of the total body surface area (TBSA)) and moderately injured (<20% TBSA burned) [24]. To limit confounding factors in the study, only samples from adult men were included. Sepsis was considered present based on criteria from the American Burn Association [25]: laboratory signs of infection (e.g., reduced platelets (not caused by bleeding), increased or reduced leucocytes, increased CRP and procalcitonin (PCT)), clinical signs of infection (e.g., body temperature >39 or <36.5 °C, signs of pneumonia, obvious wound infections), and positive bacterial cultures from blood, wounds or airways. Signs of newly developed circulatory instability were also included in the sepsis assessment, such as reduced blood pressure, increasing lactate levels and need for intravenous fluid and vasoactive support.

Daily monitoring of the degree of organ dysfunction (serum albumin levels, bilirubin and creatinine levels, platelet count, Glasgow Coma Scale score, maximal vasoactive/inotrope dose, and lowest PaO_2_/FiO_2_) were registered and Sequential Organ Failure Assessment (SOFA-score) was determined on every sampling day. Other laboratory tests (e.g., CRP, procalcitonin, leukocyte count, and microbiological cultures) were performed as clinically needed. Patients’ weight and daily cumulative fluid balance were determined on each day of sampling. Outcome (28 day and 3 month mortality) was registered for each patient.

### 2.2 Sample collection

Blood samples for analysis of steroids were collected from arterial line/venipuncture upon admission and in intervals thereafter at 1, 3, 7, 14 and 21 days after admission. Blood samples were centrifuged at 2000*g* for 10 min, plasma was separated and stored at −70 °C until analysis.

### 2.3 Steroids analysis

Five classes of steroids were measured in plasma samples using LC-MS/MS: glucocorticoids (cortisol, cortisone, corticosterone, 11-deoxycortisol); androgens (dehydroepiandrosterone (DHEA), androstenedione, testosterone); pregnanes (pregnenolone, 17-OH pregnenolone); estrogens (estrone and estradiol); and progestins (17-OH progesterone, progesterone).

Testosterone (Te), estrone (E1), estradiol (E2), pregnenolone (Pregn), cortisol, cortisone, 17-hydroxypregnenolone (17OHPregn), 17-hydroxyprogesterone (17OHP), formic acid, hydroxylamine, trifluoroacetic acid, sodium carbonate and dansyl chloride were purchased from Sigma Chemical Company (St Louis, MO, USA).

Androstenedione (A4), dehydroepiandrosterone (DHEA) and progesterone (PROG) were purchased from Steraloids Inc. (Newport, RI, USA). Deuterium labeled analogues of the steroids d_3_-testosterone, d_3_-pregnenolone, d_2_-11-deoxycortisol, d_9_-17-OH progesterone, d_3_-17OH pregnenolone, d_4_-cortisol, d_3_-cortisone, (purchased from Cambridge Isotope Laboratories, Andover, MA, USA), and d_4_-estrone and d_3_-estradiol, (purchased from CDN Isotopes, Toronto, ON, Canada) were used as internal standards. Calibration standards were prepared using standards purchased from Cerilliant (Round Rock, TX, USA). All other chemicals were of the highest purity commercially available.

Samples were analyzed as previously described [26], [27], [28], [29]. In short, steroids were extracted from 100 μL of plasma aliquots; cortisol and corticosterone were analyzed as described in [30]; DHEA, A4, Te, Pregn, 17OHPregn, 17OHP and progesterone (P4) were derivatized with hydroxylamine to form oxime derivatives; estrone and estradiol were derivatized with dansyl chloride to form dansyl derivatives [27]. Limits of quantification (LOQ) are presented in Table 1 [28]. The intra-assay and inter-assay CVs were <8% and <11%, respectively [26], [27], [28]. All steroids were analyzed in positive ion mode using an electrospray ion source on a triple quadruple mass spectrometer (AB Sciex 5500; Foster City, CA, USA). The HPLC system consisted of series 1260 and 1290 HPLC pumps (Agilent Technologies, Santa Clara, CA, USA), and an HTC PAL autosampler (LEAP Technologies, NC, USA) equipped with a fast wash station. Two mass transitions were monitored for each steroid and its internal standard (IS). Quantitative data analysis was performed using Analyst® 1.5.2 software. Calibration curves were generated with every set of samples using six calibrators. Three quality control samples were included with every set of samples. Specificity of the analysis for each steroid in every sample was evaluated by comparing concentrations determined using the primary and the secondary mass transitions of each steroid and its internal standard [31].

**Table 1:** Limits of quantification.

	Limit of quantification
Cortisol	1 ng/mL
Cortisone	1 ng/mL
Corticosterone	0.1 ng/mL
11-deoxycortisol	0.05 ng/mL
Dehydroepiandrosterone	0.05 ng/mL
Estrone	1 pg/mL
Estradiol	1 pg/mL
Pregnenolone	0.05 ng/mL
17OH-pregnenolone	0.05 ng/mL
Progesterone	1 ng/mL
Testosterone	0.01 ng/mL
Androstenedione	0.01 ng/mL
17OH-pregnenolone	0.25 ng/mL

### 2.4 Protein assay

Total protein concentration in the samples was determined using a NanoDrop™ 8000 spectrophotometer (Thermo Scientific, Wilmington, DE, USA) [32]. The optical path of the spectrophotometer was blanked, then 2 μL aliquots of each sample were loaded on optical pedestals of the instrument and total protein concentration determined using direct absorbance measurements at 280 nm. Bovine serum albumin QC samples, prepared at concentrations of 20 and 80 mg/mL and analyzed in duplicate, were analyzed along with the patient samples. In addition, ten samples out of the set were analyzed in duplicate. The imprecision of repeat measurements was <5%.

### 2.5 Statistical analysis

**Fig. 1:**
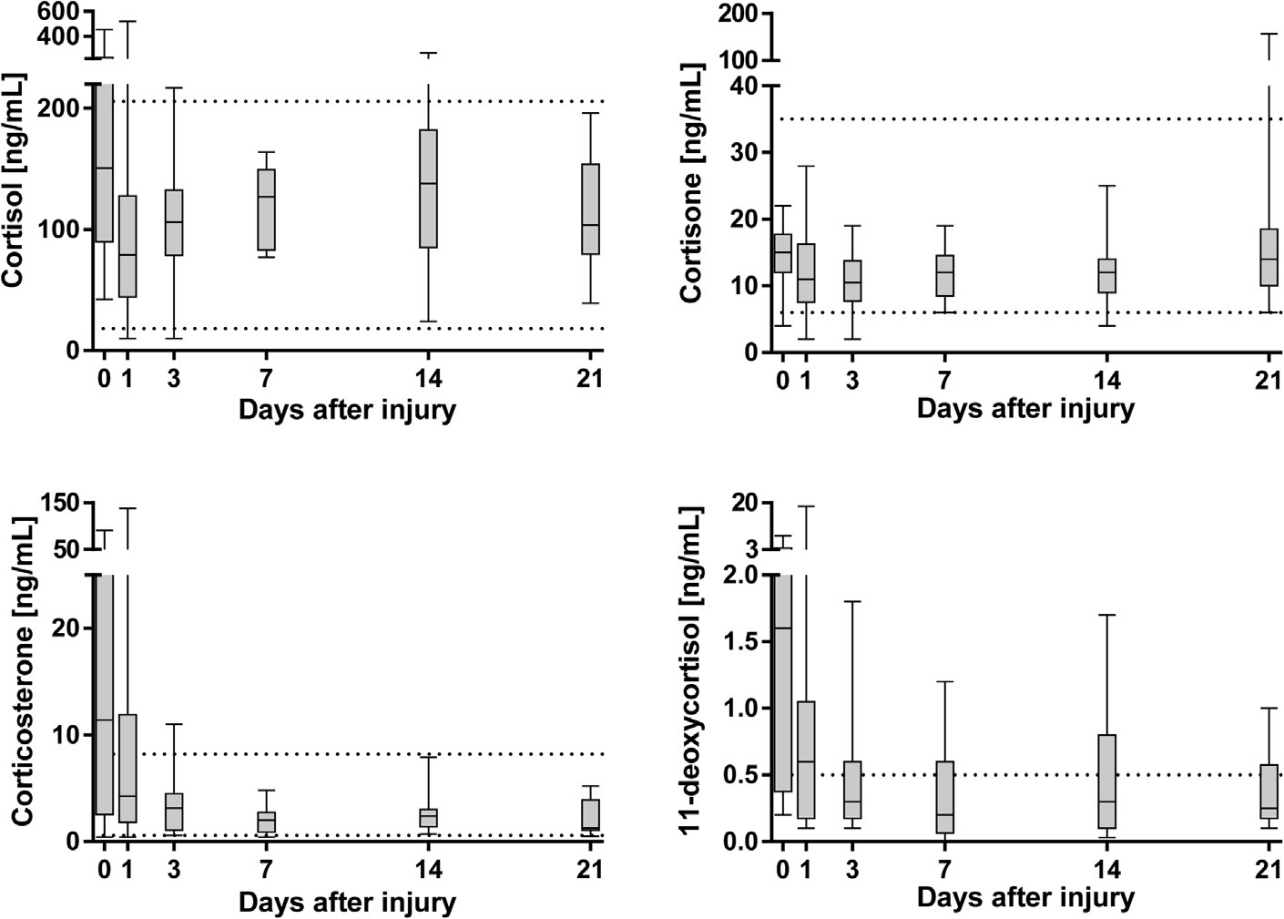
Glucocorticoid concentrations in severely and moderately burn-injured patients from admission to day 21 quantified by LC-MS/MS. Dotted lines represent reference intervals for healthy male adults. TBSA; total body surface area.

**Fig. 2:**
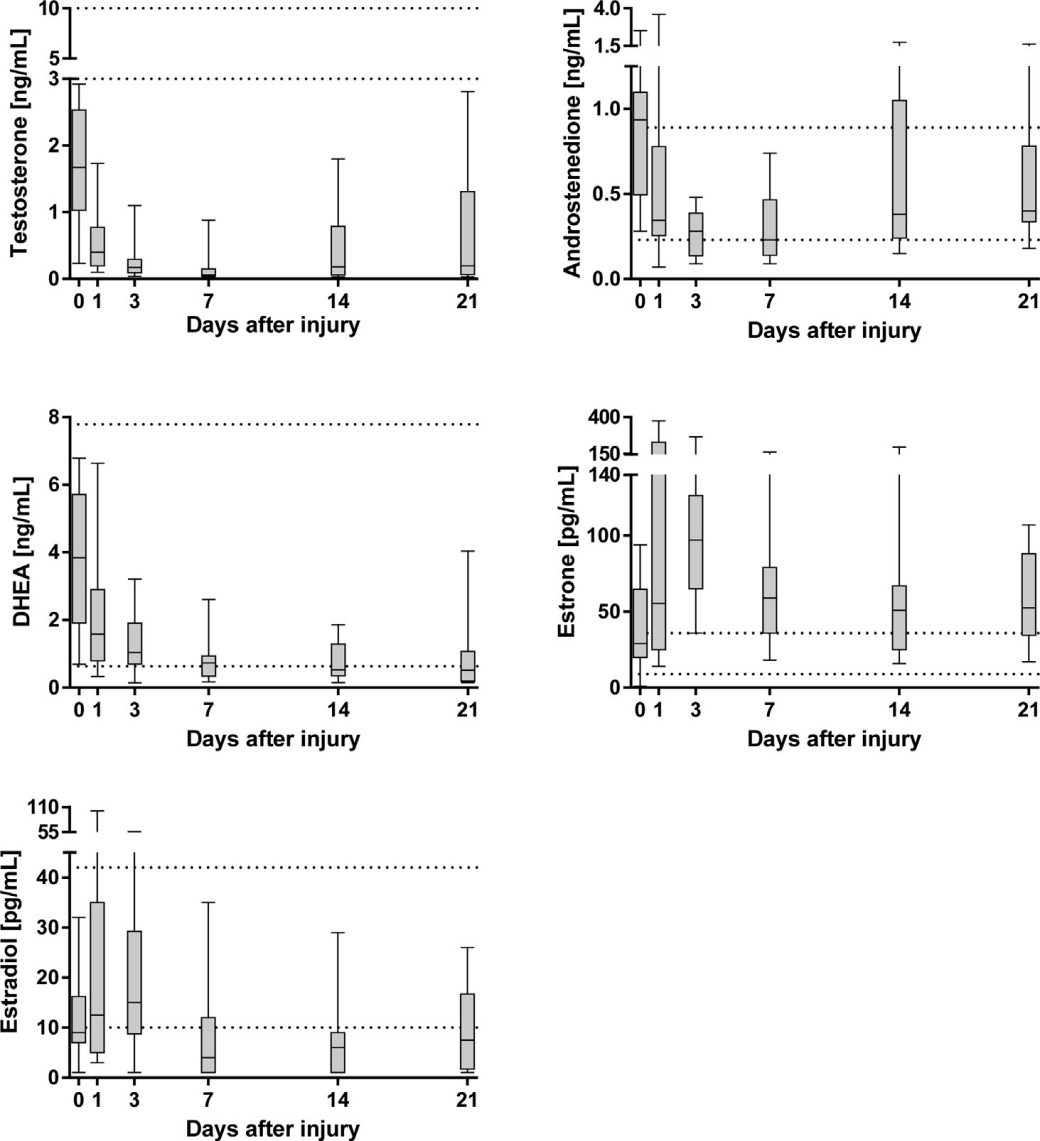
Sex steroid concentrations in severely and moderately burn-injured patients from admission to day 21 quantified by LC-MS/MS. Dotted lines represent reference intervals for healthy male adults. TBSA; total body surface area.

**Fig. 3:**
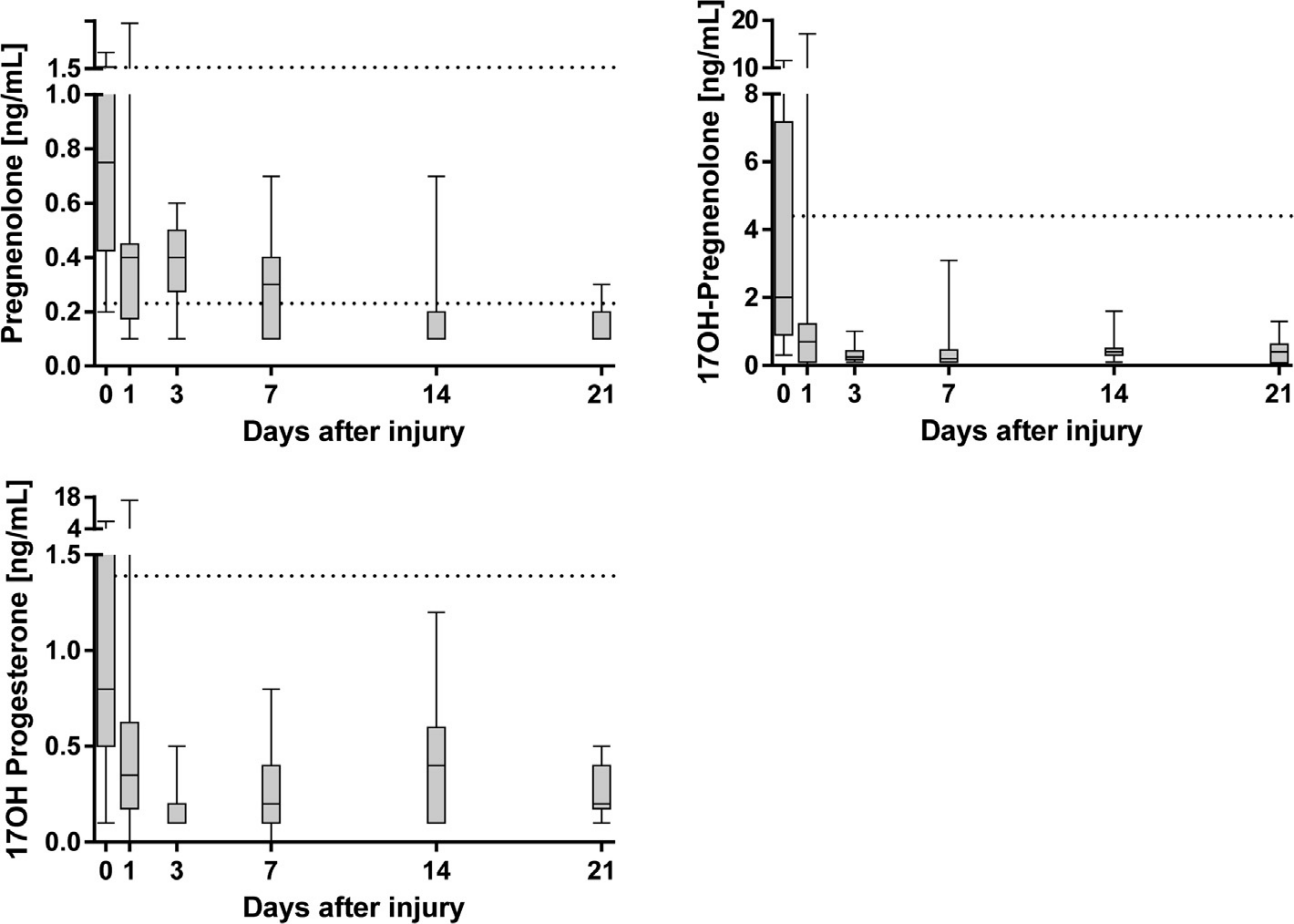
Progestagen concentrations in severely and moderately burn-injured patients from admission to day 21 quantified by LC-MS/MS. Dotted lines represent reference intervals for healthy male adults. TBSA; total body surface area.

Descriptive data are presented as boxplots and whiskers (min and max) (Fig. 1, Fig. 2, Fig. 3 and S1), or as means and standard deviation (Fig. S2). As the results for several of the measured steroids were non-normally distributed using the D’Agostino-Pearson omnibus normality test, correlations of the concentrations of steroids with burn size were assessed using the Spearman correlation test. Differences in concentration of steroids between survivors and non-survivors on day 1 after injury were as tested using a two-tailed Mann–Whitney test. *p* values < 0.05 were considered statistically significant. Statistical analysis was performed using Graphpad Prism v6.05 for Windows (Graphpad Software Inc., La Jolla, CA, USA)

## 3 Results

In this descriptive study we quantified adrenal and gonadal steroids in plasma from male adult patients with burn injury. Patient demographics are presented in Table 2. The median (range) burn surface in the sixteen patients was 32% (15–72%) of TBSA. There was no statistically significant difference in total protein or serum albumin concentrations between the severely and moderately injured (Fig. S1). The median values of the total protein and serum albumin concentration were outside of the reference intervals for healthy adults (dotted and dashed lines, Fig. S1); concentrations of total protein were the lowest on days 0–3 after the injury and increased during the recovery, while serum albumin concentrations remained below the reference intervals characteristic of healthy adults until day 21 following the injury. Over time, while the concentration of albumin tended to decrease after burn injury, the concentration of total protein tended to increase. As no significant difference was observed in adrenal and gonadal steroids between the severely and moderately injured patients, plasma steroid levels are presented as one group throughout the study.

**Table 2:** Demographics of study patients.

	Burn > 20% TBSA (n = 12)	Burn < 20% TBSA (n = 4)	p-Value
Age in years, median (range)	60 (18–69)	49 (20–75)	0.61
Body mass index, median (range)	25.0 (20.5–27.8)	25.0 (23.8–28.6)	0.41
Inhalation injury	6/12	3/4	0.42
Ventilator treated	9/12	3/4	1.0
Sepsis at any time during study period	8/12	1/4	0.17
Heart disease	3/12	2/4	0.38
Abuse	2/12	0/4	0.48
Hours from injury to first sample, median (range)	5 (2–14)	7 (6–10)	0.39
Survival 21 days	10/12	3/4	0.73
Survival 3 months	10/12	3/4	0.73

TBSA, total body surface area.

### 3.1 Glucocorticoids

**Fig. 4:**
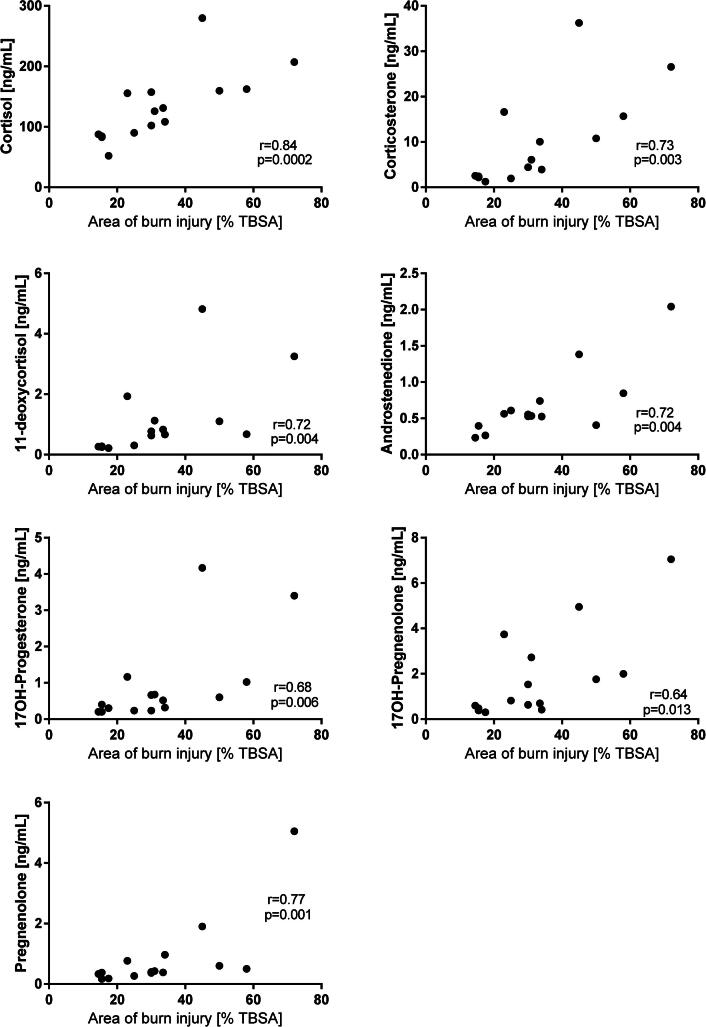
Scatter plots representing associations between endogenous steroid concentrations and area of burn injury in all patients at admission. Correlations were analyzed using Spearman correlation test.

In most patients with burn injury, concentrations of cortisol, corticosterone and 11-deoxycortisol were highest upon admission to the burn center (Fig. 1). Maximum concentrations of cortisone were observed on day 21 after burn injury in several patients. Upon admission, concentrations of corticosterone and 11-deoxycortisol were above reference values for healthy adult men among the patients (0.60–8.20 ng/mL and <0.5 ng/mL, respectively). Concentrations of cortisol, corticosterone and 11-deoxycortisol were found to correlate with the area of burn injury (Fig. 4). Concentrations of cortisone were higher in non-survivors compared to survivors on day 1 after the injury (Fig. S2, *p* = 0.006).

### 3.2 Sex steroids

In all patients, the median concentrations of testosterone, androstenedione and DHEA were highest upon admission to the burn center (Fig. 2). The testosterone concentrations decreased during the acute phase and were up to ten times lower than the reference values expected in healthy adult men (3.0–10.8 ng/mL) [29]; the concentrations did not recover during the study period (Fig. 2). At the end of the study period, testosterone concentrations increased in patients of younger age (<50 years, Fig. S3), while remaining below the values characteristic of healthy adult men. Patients of older age (>50 years) did not display an increase in testosterone concentration during the study period. Concentrations of estradiol were below the reference intervals expected of healthy adult men (10–42 pg/mL) [28] between day 7 after injury until the end of the study period. In contrast, patients displayed elevated concentrations of estrone, with values above the reference intervals characteristic of healthy adult men (9–36 pg/mL [28], Fig. 2). During the study period, concentrations of estrone did not retrocede to the values expected of healthy men. Contrary to the other steroids, the highest concentrations of estrone were observed on day 3 after injury and then declined by day 21 to concentrations comparable to those observed on the day of the injury. In most of the patients, concentrations of estradiol were the highest on day 3 after injury. Concentrations of testosterone and estradiol did not correlate with the size of the injury, while estrone displayed a nearly significant correlation with the area of burn injury (*r* = 0.490, *p* = 0.065, data not shown). Concentrations of androstenedione and DHEA were higher in the non-survivors compared to survivors on day 1 after the injury (Fig. S2, *p* = 0.011 and 0.006, respectively).

### 3.3 Progestagens

In patients with burn injury, concentrations of pregnenolone, 17OH-pregnenolone and 17OH-progesterone were the highest upon admission to the burn center (Fig. 3). Upon admission, concentrations of 17OH-pregnenolone were above the reference values for healthy adult men (<4.42 ng/mL). In contrast, 21 days after injury, patients displayed median concentrations of pregnenolone below the reference values for healthy adult men (0.23–1.73 ng/mL) [29]. Concentrations of pregnenolone, 17OH-pregnenolone and 17OH-progesterone were found to correlate to the area of burn injury (Fig. 4). Concentrations of pregnenolone, 17OH-pregnenolone and 17OH-progesterone were higher in the non-survivors compared to survivors on day 1 after injury (Fig. S2, *p* = 0.036, 0.019 and 0.008, respectively).

## 4 Discussion

Burn injury causes an extreme increase in metabolic rate and energy requirements, with the changes affecting the rate of the biosynthesis of endogenous steroids [6]. In this study we observed that concentrations of cortisol, corticosterone, 11-deoxycortisol, androstenedione, pregnenolone, 17OH-pregnenolone, and 17OH-progesterone were associated with the size of burn injury in male patients, suggesting that concentrations of circulating endogenous adrenal and gonadal steroids are affected by burn injury. In detail, we observed that cortisol plasma levels correlated positively with the burn injury size, despite being within the reference intervals in most patients.

Elevated concentrations of plasma cortisol are known to be a hallmark of critical illness [33], [34], and inappropriately low concentrations of cortisol have previously been linked to an increased mortality [35]. It is well known that burn injury disturbs the circadian rhythm and diurnal pattern of circulating plasma cortisol [8], [36], [37]. The release of cortisol and DHEA are both responses to the level of adrenocorticotrophic hormone (ACTH), and are both potent modulators of the immune response. Our results, which demonstrate a combination of elevated concentrations of cortisol and decreased concentrations of DHEA, are in agreement with previously reported findings following thermal injury [7], [38]. In the present study, we observed increased concentrations of estrogens and decreased concentrations of androgens, which have also been observed in other severe conditions, such as sepsis, shock and myocardial infarction [39], [40], [41], [42].

In patients with burn injury, low concentrations of testosterone have been associated with osteoporosis, loss of lean body mass and impaired wound healing [43]. As testosterone is the main endogenous anabolic steroid, hypotestosteronemia following critical illness may have severe ramifications for the patient, such as decreased lean body mass, muscle function impairment and exercise intolerance [44]. Thus, hypotestosteronemia in burn-injured patients may increase the risk of delayed ventilator weaning and physical recovery, although we did not investigate these parameters in this study. Furthermore, testosterone has been shown to have immune-depressive effects in trauma-hemorrhage in mice, increasing the susceptibility to sepsis and even worsening the outcome after sepsis [45], [46].

**Fig. 5:**
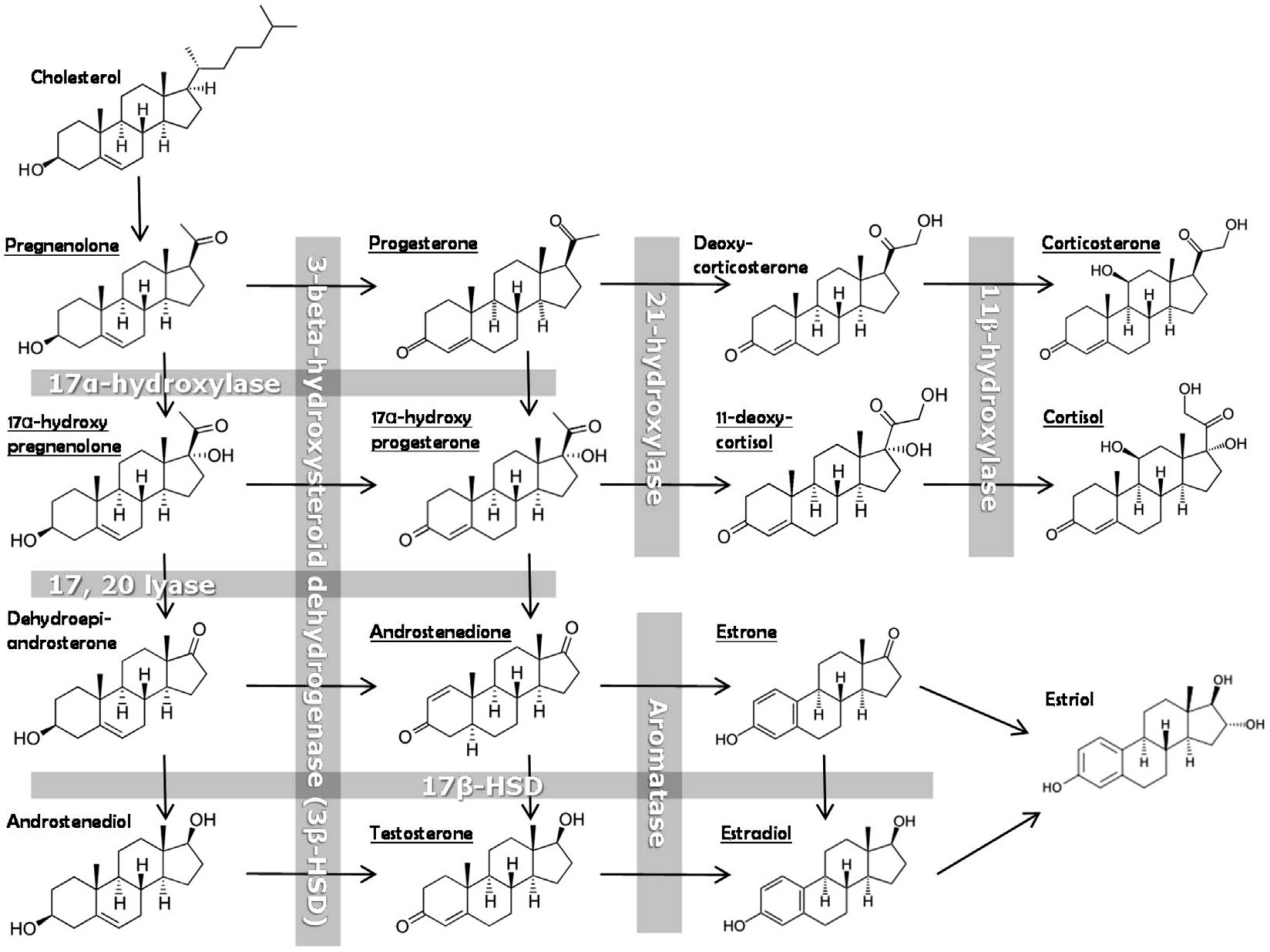
Schematic overview of the steroid pathway. The obtained results in this study suggest a general reduction of the 17β-HSD enzymatic pathway resulting in decreased concentrations of androstenedione, testosterone and estradiol, and increased activity of aromatase, leading to increased concentrations of estrone.

Low concentrations of circulating testosterone following burn injury may potentially be caused by several factors, such as downregulated hypothalamic-pituitary–gonadal (HPG) axis, effect of cytokines on Leydig cells, and increased peripheral aromatase activity. Aromatase is required for biosynthesis of estrone and estradiol and is present in several tissues, such as skin, bone, adipose tissue, arteries and brain [47]. Increased aromatase activity following burn injury likely contributes to the elevated concentrations of estrone observed in patients in this study (Fig. 5). Elevated concentrations of estrone have previously been correlated with severity and outcome in septic shock [48]. Estrone has also been shown to be neuroprotective in rats following traumatic brain injury (TBI) and may stimulate neurogenesis [49]. The increased levels of estrone observed in this study and increased tolerance to a burn injury, could be explained in part to arrested conversion of androstenedione to testosterone by 17β-hydroxysteroid hydrogenase (17β-HSD), in favor of estrone biosynthesis by action of aromatase (Fig. 5).

Recently, it was shown that estradiol administration in rats following scald burn had a protective effect by attenuating the hypermetabolic state [50], while testosterone depletion by castration was shown to protect mice from heat-induced multi-organ damage and lethality [51]. Moreover, high testosterone concentrations may lead to immunologic impairment as a direct effect of alteration of cell-mediated immunity and cytokine expression [52], which further supports the theory that the increased levels of estrone observed in patients in the present study, could have a survival benefit in burn injury. Inhibition of 17β-HSD and/or activation of aromatase, leading to a shift from androgen to estrogen hormonal responses, in turn would conserve energy for essential metabolism and wound healing instead of building muscles and bone.

An increased plasma estrone concentration is likely to reflect a transiently elevated peripheral aromatization in adipose tissue, associated with the burn injury. This is further supported by alterations in adipose tissue observed in response to critical illness, such as increased number of adipocytes [53]. In addition, estrogen administration in rats after severe scald injury attenuated the hypermetabolic losses of body mass [50], which further supports that the hormonal shift towards estrogens in patients with burn injury may be an adaptive mechanism of burn trauma, rather than an association with a poor prognosis.

In evaluation of the data presented it should be noted that only male patients were enrolled since the estrus cycle in female patients, which induces fluctuations in circulating adrenal and gonadal steroids, would have introduced a confounding factor. Furthermore, the time at which blood was sampled from patients, after admission and inclusion in the study, was not consistent across the time of day. As both adrenal and gonadal hormones are known to have a circadian rhythm, this could be considered a caveat, however, the diurnal pattern has been reported to be abolished after burn injury [8], [36], [37], and, hence, was not expected to affect our findings. Direct comparison between age groups was not possible because of the age differential between the severely and moderately injured groups.

While associations between several endogenous steroids and burn size were identified, as well as a survivor / non-survivor bias between some steroids, both observations require a larger sample size for confirmation. Additionally, it should be noted that reference values from healthy men should be compared with burn injured patients with caution, as patients with critical illness often suffer from other pathophysiological differences as well as major changes in vascular permeability [54]. In turn, vascular leakage could thus cause variations in total protein, as well as serum albumin and steroid-binding globulin concentrations, which is likely to affect the concentration of free and bioavailable steroids. As albumin is a negative acute-phase reactant protein (ARP) and immunoglobulins are positive ARP, this could explain the observed opposite trends of the changes in concentrations of albumin and total protein. Our study does not aim to explain the causal mechanism for the altered steroid synthesis in burn injury, but does support a relationship.

## 5 Conclusion

Our data indicate that burn injury alters endogenous steroid biosynthesis, with decreased testosterone concentrations and elevated estrone concentrations during the first 21 days after the injury. Concentrations of plasma cortisol, corticosterone, 11-deoxycortisol, androstenedione, 17OH-progesterone, 17OH-pregnenolone and pregnenolone were found to correlate with the area of burn injury. Concentrations of testosterone decreased during the acute phase, were up to ten-times lower than the reference values expected in healthy adult men, and did not recover during the study period. By day 21 after injury, testosterone concentrations increased in patients of younger age, but not in older patients.

The highest concentrations of estrone were observed on day 3 after injury and then declined by day 21 to concentrations comparable to those observed on the day of injury. No difference in the concentration of steroids was observed between patients with severe and moderate burn injury. If the finding of elevated estrone concentrations can be confirmed in a larger hypothesis-driven study, this may have a future therapeutic potential. Further studies will be needed to delineate the underlying mechanisms behind alterations in steroid biosynthesis after burn injury.

## Competing interests

The authors declare to have no competing interests.

## Appendix A Supplementary data

Supplementary data 1 Fig. S1. Distribution of total protein and serum albumin concentration over time from admission to day 21 in burn injured patients. Dotted lines represent serum albumin, and dashed lines represent total protein reference intervals for healthy adults. TBSA; total body surface area. Fig. S2. Bar graphs (mean and standard deviation) of steroids in burn injured male patients on day 1 after injury. Differences between survivors and non-survivors were tested using two-tailed Mann-Whitney test. ^*^*p* < 0.05, ^**^*p* < 0.01. Fig. S3. Box plots with distributions of testosterone concentration in the patients over time from admission to day 21. A, patients younger than 40 years of age; B, patients older than 40 years of age. Dotted lines represent the low boundary reference intervals for healthy men.
