# Supplementary figures and images for: Altered adrenal and gonadal steroids biosynthesis in patients with burn injury
Source: Clin Mass Spectrom. 2016 Oct 26;1:19–26. doi: 10.1016/j.clinms.2016.10.002 (PMC11324613; doi:10.1016/j.clinms.2016.10.002)

Fig S1

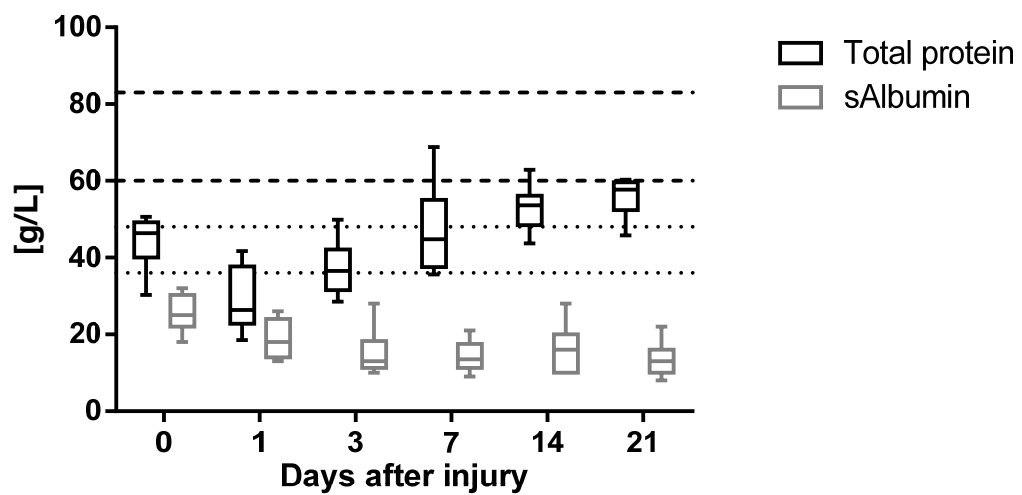

Fig S2

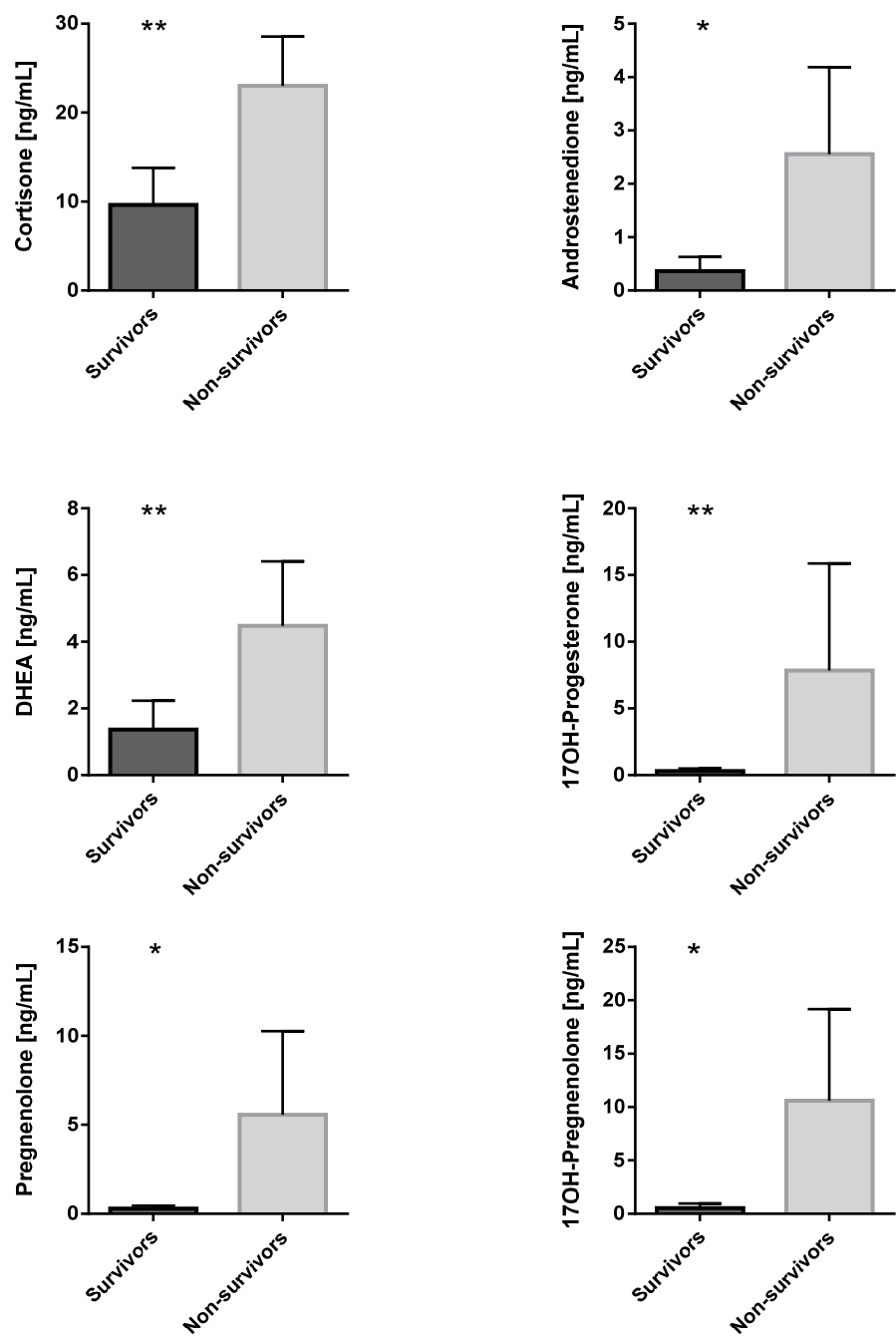

Fig S3

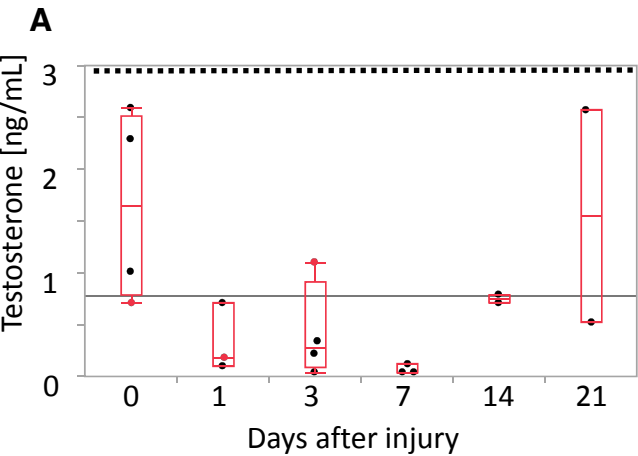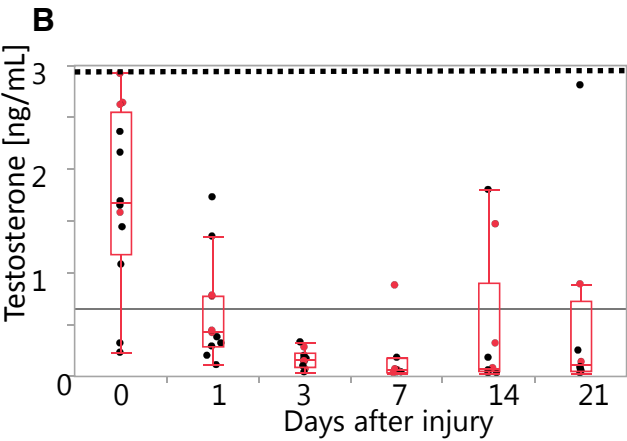

Supplement: Supplementary data 1 — Fig. S1. Distribution of total protein and serum albumin concentration over time from admission to day 21 in burn injured patients. Dotted lines represent serum albumin, and dashed lines represent total protein reference intervals for healthy adults. TBSA; total body surface area. Fig. S2. Bar graphs (mean and standard deviation) of steroids in burn injured male patients on day 1 after injury. Differences between survivors and non-survivors were tested using two-tailed Mann-Whitney test. *p < 0.05, **p < 0.01. Fig. S3. Box plots with distributions of testosterone concentration in the patients over time from admission to day 21. A, patients younger than 40 years of age; B, patients older than 40 years of age. Dotted lines represent the low boundary reference intervals for healthy men. [file mmc1.pdf]
